# Immunotherapy in the Treatment of Undifferentiated Pleomorphic Sarcoma and Myxofibrosarcoma

**DOI:** 10.1007/s11864-025-01349-x

**Published:** 2025-09-01

**Authors:** Jerry T. Wu, Elizabeth Nowak, Jarrell Imamura, Jessica Leng, Dale Shepard, Shauna R. Campbell, Jacob Scott, Lukas Nystrom, Nathan Mesko, Gary K. Schwartz, Zachary D. C. Burke

**Affiliations:** 1 Cleveland Clinic Lerner College of Medicine of Case Western Reserve University, Cleveland, OH USA; 2 Genomic Medicine Institute, Cleveland Clinic Research, Cleveland Clinic, Cleveland, OH USA; 3 Case Western Reserve University School of Medicine, Cleveland, OH USA; 4 Department of Hematology and Oncology, Taussig Cancer Institute, Cleveland Clinic, Cleveland, OH USA; 5 Department of Radiation Oncology, Taussig Cancer Institute, Cleveland Clinic, Cleveland, OH USA; 6 Department of Orthopedic Surgery, Cleveland Clinic, Cleveland, OH USA; 7 Case Comprehensive Cancer Center, Cleveland, OH USA; 8 Department of Translational Hematology and Oncology Research, Lerner Research Institute, Cleveland Clinic, Cleveland, OH USA

**Keywords:** Sarcoma, Undifferentiated pleomorphic sarcoma, Myxofibrosarcoma, Immunotherapy, Tumor microenvironment, Biomarkers

## Abstract

Undifferentiated pleomorphic sarcoma (UPS) and myxofibrosarcoma (MFS) are among the most common adult soft tissue sarcoma (STS) subtypes. Due to their high genetic complexity, heterogeneity, and lack of specific genetic alterations, no consistent molecular targets for targeted therapy have been identified for UPS and MFS. Recently, immune checkpoint inhibition (ICI) has emerged as a promising treatment modality for UPS and MFS. However, the efficacy of ICI in UPS and MFS remains far lower than in other cancers such as melanoma. Strategies to increase the efficacy of ICI, including selecting patients based on putative biomarkers and combining ICI with chemotherapy, targeted therapies, and/or radiation therapy, are currently in clinical development. In this review, we first summarize the clinical characteristics of UPS and MFS, examining the tumor microenvironment (TME) and its effect on the efficacy of ICI. We then review putative biomarkers of ICI response and highlight clinical trials testing ICI in patients with UPS and MFS. Finally, we discuss other forms of immunotherapy for UPS and MFS currently under preclinical investigation. The combination of ICI plus radiation therapy appears to have benefit for patients with localized UPS and MFS. ICI should be considered for patients with advanced or unresectable UPS and MFS, especially those with potential biomarkers of response such as tertiary lymphoid structures (TLS). However, singular biomarkers such as TLS may prove inadequate to predict ICI response; more accurate prediction will likely require a panel of biomarkers including TLS, immune cell infiltration, PD-L1 expression, and other TME components.

## Introduction

Undifferentiated pleomorphic sarcoma (UPS) and myxofibrosarcoma (MFS) are among the most common adult soft tissue sarcoma (STS) subtypes, comprising 14% and 5% of adult STSs respectively [1, 2]. Although UPS and MFS are classified as distinct clinical entities based on differing clinicopathologic features – with MFS having prominent myxoid stroma and UPS generally being higher grade and more prone to distant metastasis – genomic and transcriptomic profiling have revealed the two STS subtypes to be largely indistinguishable [3, 4]. Given their similarity, common treatment approaches for both subtypes may be appropriate. Standard of care for non-mestatic UPS and MFS includes complete resection and, for intermediate- and high-grade tumors, radiation therapy. The role of systemic therapy in these tumors has been uncertain. Immunotherapy has dramatically improved outcomes for patients with many common types of cancer such as breast carcinoma, melanoma, and lung carcinoma [5]. However, these therapies have shown limited efficacy in UPS and MFS until recently. Recent clinical trials of immunotherapy have demonstrated remarkable efficacy in STS, including UPS and MFS [6–9]. In this review, we discuss the tumor microenvironment (TME) and TME biomarkers that potentially predict response to immunotherapy. Furthermore, we review the existing literature on the use of immunotherapy in UPS and MFS.

## Clinical Characteristics of UPS and MFS

UPS and MFS predominately affect adult patients, with an average age of presentation between 60 and 70 years, and typically arise in the trunk or extremity [2, 10, 11]. Both subtypes are highly aggressive, with a 5-year survival rate of between 60 and 70% [12–14]. In patients who develop metastatic disease, survival is poor, with a 5-year survival reported at 20% [12, 14–17].

Diagnosis of UPS and MFS requires biopsy and histologic evaluation by an expert STS pathologist [11]. Morphologically, UPS is characterized by marked cellular pleomorphism, spindle-shaped cells, nuclear atypia and hyperchromasia, a lack of differentiation, and a disorganized or storiform cellular arrangement [18, 19]. MFS is characterized by a variable amount of myxoid stroma, cellular pleomorphism, and a distinctive curvilinear vascular pattern [20]. MFS tumors can range from low- to high-grade, whereas UPS is almost uniformly high-grade. MFS has a predilection for superficial locations, but UPS and MFS can occur in both superficial and deep locations. Distinguishing between UPS and MFS – especially higher grade MFS tumors – is often challenging due to the similarities in morphology and the lack of molecular markers specific to either subtype [18]. Lee et al. found that increased myxoid content in MFS is associated with improved survival, and suggested that a cutoff of 5% myxoid component threshold could help differentiate UPS and MFS for clinical decision-making; however, this criterion has yet to be widely adopted [14, 18].

Current treatment for UPS and MFS relies on an oncologic (complete) surgical resection with the goal of achieving negative margins. Surgery is often combined with adjuvant radiation therapy and/or chemotherapy [21, 22]. Neoadjuvant or adjuvant radiation therapy is often recommended to improve local control in high-grade tumors, especially in cases where achieving clear surgical margins is difficult. For patients with high-risk features or metastatic disease, systemic chemotherapy may be considered, although its benefit is not universally accepted. No specific regimen for UPS or MFS has been developed; the first choice chemotherapy for UPS and MFS is the AIM regimen (doxorubicin, ifosfamide, and mesna), which is recommended for all adult STSs [1, 21]. However, response rates are variable and the regimen is associated with numerous toxicities [23, 24]. As such, the role and proper application of chemotherapy in UPS/MFS remain unresolved [25]. Novel therapeutics that improve survival and reduce toxicities for patients with UPS and MFS are greatly needed.

The development of these therapeutics has faced many challenges. UPS and MFS are complex karyotype sarcomas, which are associated with a heterogenous array of genetic and chromosomal abnormalities including losses, gains, amplifications, and point mutations, while lacking specific driver mutations or recurrent genetic translocations seen in simple karyotype sarcomas [26]. This high genetic complexity, combined with heterogeneity and a lack of specific genetic alterations, has made it difficult to identify consistent molecular targets for targeted therapy in UPS and MFS. Additionally, UPS and MFS have historically been considered immunologically “cold” tumors, with low levels of immune cell infiltration, high levels of immunosuppressive cells, and relatively low tumor mutation burden compared to other cancers, which potentially limit the effectiveness of immunotherapies [27–29]. Until recently, immunotherapies have shown disappointing results in the setting of advanced UPS and MFS, but recent trials are beginning to demonstrate improved response rates in selected patients and in combination with radiation, chemotherapy, and/or immunomodulatory agents [9, 30, 31].

## TME of UPS and MFS

### Tumor mutational burden (TMB) and copy number alteration (CNA)

TMB is defined as the number of somatic mutations per coding area within a tumor’s genome [32]. It serves as a proxy for neoantigen burden, which can influence the immune system’s ability to recognize and attack cancer cells [33, 34]. STSs, although a heterogenous group of tumors, have been shown as a whole to harbor a low TMB, with a median of 2.5 mutations/Mb and only 5% of tumors harboring > 20 mutations/Mb (compared to basal cell carcinoma, which has a median of 47.3 mutations/Mb and 70.7% of tumors harboring > 20 mutations/Mb) [35]. This is true of UPS and MFS, with a median TMB of 2.5 and 2.2 mutations/Mb respectively [35]. UPS and MFS are characterized by a high number of CNA, which are defined as variations in the number of copies of particular genes and can theoretically increase neoantigen formation [3]. However, CNA are considered to be less immunogenic than mutations [36]. The utility of TMB as a prognostic tool in UPS is debated. Higher TMB was found to trend with improved overall survival (OS) in STS in a study by Raj et al., although this was nonsignificant (*p*= 0.08) [37]. Higher TMB was found to trend with increased immune checkpoint inhibition (ICI) response rate in STS in a study by Lee et al., but this was also nonsignificant (*p*= 0.20) [38]. Subtypes with low TMB, such as UPS and MFS, have demonstrated relatively high response rates to ICI [38]. Taken together, TMB alone does not appear to be a clinically useful biomarker for ICI response in UPS/MFS.

### Expression of Programmed Death-ligand 1 (PD-L1)

PD-L1 is a transmembrane protein that interacts with the programmed death-1 (PD1) receptor on T cells and other immune cells to inhibit T cell activity, promote immune tolerance, and prevent autoimmunity [39]. PD-L1 is commonly overexpressed on cancer cells and in the TME, thereby allowing cancer cells to evade immune surveillance. In the context of STS, high PD-L1 expression is generally associated with higher metastatic potential and worse outcome [40–42]. Studies in a variety of cancers have demonstrated positive correlation between PD-L1 expression and response to ICI, although this correlation remains debated in sarcoma [43]. In STS, PD-L1 positivity is commonly defined as ≥ 1% tumor cells expressing PD-L1 [42, 44]. Various studies have reported that the rate of PD-L1 positivity in UPS ranges from 23 to 73% [25, 29, 42, 45, 46]. In a study by Lee et al., of PD-L1 positive UPS tumors, 3.5% of tumors were found to have 1–4% of tumor cells expressing PD-L1, 24% of tumors had 5–9% of tumor cells expressing PD-L1, 27% of tumors had 10–49% of tumor cells expressing PD-L1, and 18% of tumors had ≥ 50% of tumor cells expressing PD-L1 [25]. Per Boxberg et al., Vargas et al., and Pollack et al., UPS has the highest prevalence of PD-L1 positivity of all STS [29, 45, 47]. Regarding MFS, studies have reported PD-L1 positivity in 16% − 35.6% of tumors [45, 48, 49]. In a study by Wunder et al., 48% of MFS tumors were found to have ≤ 5% of tumor cells expressing PD-L1, 12% had 5–20% of tumor cells expressing PD-L1, 20% had 20–30% of tumor cells expressing PD-L1, 12% had 30–50% of tumor cells expressing PD-L1, and 8% had ≥ 50% of tumor cells expressing PD-L1 [49]. Interestingly, several studies that looked at specifically at UPS have reported that there is a positive association between PD-L1 expression and improved survival, contrary to previous findings that PD-L1 expression is associated with worse outcomes [25, 29, 49]. Additionally, several studies have shown that PD-L1 expression correlates positively with response to ICI in STS, with a large retrospective study finding that STS with PD-L1 ≥ 1% were significantly more likely to respond to ICI than those with PD-L1 < 1% (*p*= 0.02) [38, 50, 51]. Nevertheless, PD-L1 expression alone does not appear to be a clinically useful biomarker for predicting response to ICI as responses are also frequently seen in PD-L1 negative STS.

### Immune Cell Infiltration

Tumor-infiltrating immune cells (TIIC) are a critical component of the TME and play diverse roles in tumor progression and response to therapy. TIIC include various immune cell types such as CD8 + T cells, natural killer (NK) cells, regulatory T cells (Tregs), myeloid-derived suppressor cells (MDSCs), and tumor-associated macrophages (TAMs) [5]. CD8 + T cells and NK cells are primarily involved in anti-tumor responses, with high densities of these cells generally associated with improved patient outcomes. Conversely, Tregs, MDSCs, and M2-polarized macrophages are thought to promote tumor growth by suppressing the anti-tumor immune response, leading to worse patient outcomes.

Tumor associated macrophages are the most abundant cells in the UPS TME, where they are likely to be M2-polarized [52, 53]. M2 macrophages differentiate from M0 macrophages in the presence of M-CSF, IL-4, or IL-10 and express immunosuppressive cytokines PD-L1, IL-10 and TGFβ [54, 55]. The presence of M2 macrophages is thought to sustain an immunosuppressive TME that promotes tumor growth and proliferation. Among STS, UPS has the highest proportion of M2 to total macrophages, and this has been postulated to reduce the efficacy of ICI therapy [56]. 

Despite the abundance of immunosuppressive TAMs, UPS commonly has extensive T-cell infiltration, oligoclonal T-cell repertoires, and increased expression of genes involved in antigen presentation and T-cell mediated apoptosis [27, 57, 58]. Increased CD8 + T cell density has been negatively correlated with tumor size and positively correlated with OS in UPS [27, 59, 60]. However, Dancsok et al. show that, despite the presence of tumor infiltrating lymphocytes (TILs) in UPS and MFS tumors, a majority of these tumors have ≥ 1 TIL that express immune checkpoint proteins LAG3, TIM-3, and PD-1, which potentially contribute to an immunosuppressive TME [58]. Moreover, Que et al. show that 36% of UPS tumors are infiltrated with FOXP3 + Tregs, which correlates with PD-L1 expression, suggesting that PD-L1 and Tregs synergistically promote UPS immune evasion [61]. Interestingly, Guegan et al. show that despite predicting a better prognosis, high immune cell infiltration, particularly of CD8 + T cells and CD20 + B cells, predicts a poor response to neoadjuvant chemotherapy in UPS [62]. They hypothesize that this nonresponse may be mediated by M2 macrophages, which are enriched alongside CD8 + T cells and CD20 + B cells.

Subramanian et al. discovered, through a machine-learning framework using bulk transcriptomes, that a group of STS termed sarcoma ecotype 3 (SE3) with a TME consisting of an intermediate level of immune infiltration characterized by M2-like immunosuppressive macrophages, MYC/MTORC1-activated epithelial-like malignant cells, mature dendritic cells, and pro-inflammatory neutrophils had the best response ICI [63]. Interestingly, they noted that UPS tumors had the highest proportion assigned to SE3, which possibly explains the high clinical activity of ICI in UPS. Additionally, they found that SE3 abundance outperformed PD-L1 expression and presence of tertiary lymphoid structures as a biomarker for predicting ICI response and 6-month non-progression, potentially enabling better identification of STS patients that could benefit from ICI therapy.

### Tertiary Lymphoid Structures (TLS)

TLS are ectopic lymphoid aggregates that form in non-lymphoid tissues at sites of chronic inflammation, including tumors [64, 65]. They resemble secondary lymphoid organs in their organization and function, containing B-cell follicles, T-cell zones, high endothelial venules, and follicular dendritic cells [66]. The presence of TLS within tumors is hypothesized to prime T cells and B cells with tumor-specific antigens. This process drives the expansion and differentiation of naive T and B cells into cytotoxic effector T cells capable of lysing tumor cells and B cells that express high-affinity antibodies for tumor antigens, respectively [64]. Moreover, TLS have been shown as a prognostic marker independent of ICI therapy and a predictive biomarker of ICI response in a range of cancers including non-small cell lung cancer, bladder cancer, gastrointestinal cancers, head and neck carcinomas, renal carcinoma, breast carcinoma, melanoma, and STSs [30, 66–71]. In STS, Petitprez et al. discovered that TLS are associated with an highly immune-infiltrated subtype of tumors with improved survival and ICI response [31]. Retrospective analysis of the SARC028 study, a phase 2 clinical trial of pembrolizumab in patients with metastatic or unresectable locally advanced STS and bone sarcoma, found that patients with STS categorized to this immune subtype had a 50% overall response rate (ORR) to ICI [8, 31, 72].

On the basis of this discovery, the PEMBROSARC study, a phase 2 clinical trial of pembrolizumab combined with cyclophosphamide in patients with advanced STSs, was amended to include a new cohort selected based on the presence of TLS [30, 56]. The goal of this modification was to investigate the efficacy of ICI therapy in patients selected for the presence of this biomarker. In the selected cohort, the 6-month non-progression rate (NPR) was 40% and overall response rate ORR was 30%, which were significantly higher than the unselected cohort, which had a 5-month NPR and ORR of 4.9% and 2.4% respectively. Surprisingly, among STSs in the selected cohort, UPS and MFS were relatively poorly responding STS subtypes, with no patients experiencing complete or partial response, 1 UPS patient and 1 MFS patient experiencing stable disease, and 3 UPS patients experiencing progressive disease (compared to dedifferentiated liposarcoma (DDLPS), which had 5 patients with PR, 6 with stable disease, and 1 with progressive disease) [30]. Nevertheless, this study found that TLS can be used to predict ICI response in patients with STS and select for patients who are more likely to respond. This finding was supported by the results from a phase 2 clinical trial by Roland et al., which tested neoadjuvant nivolumab or nivolumab/ipilimumab in patients with resectable DDLPS and UPS (the UPS cohort also received concurrent nivolumab/radiation therapy) [73]. This study found that ICI therapy increased TLS signature in DDLPS but not UPS. Additionally, the presence of TLS at surgery for DDLPS and at baseline for UPS was associated with improved OS. Of note, 2 UPS patients with TLS at baseline were found to have lost their TLS upon neoadjuvant therapy, which was hypothesized to be due to the radiosensitivity of B cells. These thought-provoking results highlight the complexity of the interactions between ICI, radiotherapy, the TME, and TLS in STS. Please refer to Table 1 for a selected list of studies of the TME of UPS/MFS.

**Table 1:** Select studies of the UPS/MFS TME

TME component	Reference	Year	Type of study	Population	Summary of findings
TMB	Chalmers et al. [35]	2017	Retrospective	102,292 samples from various cancers	STS including UPS and MFS have low TMB
	Raj et al. [37]	2022	Retrospective	259 STS from The Cancer Genome Atlas	Higher TMB trended toward an association with improved survival (*p* = 0.08)
	Lee et al. [38]	2025	Retrospective	216 patients with advanced sarcoma treated with ICI at a single institute	Higher TMB trended toward an association with increased ICI response rate (*p* = 0.20)
PD-L1	D’Angelo et al. [44]	2015	Retrospective	50 STS samples from a single institute	No association between OS and PD-L1 expression
	Kim et al. [41]	2016	Retrospective	82 STS samples from a single institute	STS patients with PD-L1 expression had worse OS than those without PD-L1 expression
	Bertucci et al. [40]	2017	Retrospective	758 STS samples from 8 public databases	High PD-L1 expression correlated with shorter metastasis-free survival
	Boxberg et al. [29]	2017	Retrospective	128 STS patients from a single institute	Positive PD-L1 expression was associated with improved OS in UPS patients
	Orth et al. [42]	2020	Retrospective	114 STS samples from a single institute	PD-L1 expression was associated with more PD-1 positive TILs, higher tumor grading, and worse OS
	Wunder et al. [49]	2020	Retrospective	226 STS and osteosarcoma patients form multiple institutes	High PD-L1 expression was associated with improved survival in UPS but not MFS
	Lee et al. [25]	2020	Retrospective	205 UPS patients from a single institute	Higher PD-L1 expression trended toward an association with improved DFS (*p* = 0.086)
	Keung et al. [50]	2020	Correlative analysis of a phase II clinical trial	86 patients with metastatic or unresectable sarcoma	Few sarcomas expressed PD-L1 at baseline. PD-L1 expression on tumor associated macrophages was associated with ICI response
	Italiano et al. [51]	2020	Pooled analysis of clinical trials of PD1 or PD-L1 antagonist	384 STS patients treated with ICI from multiple studies	There is a low proportion of patients with PD-L1 positivity. Both PD-L1 positive and PD-L1 negative patients responded to ICI, showing its limitation as a biomarker
	Yamashita et al. [48]	2022	Retrospective	45 MFS patients form a single institute	Positive PD-L1 expression was associated with a higher density of TILs
	Lee et al. [38]	2025	Retrospective	216 patients with advanced sarcoma treated with ICI at a single institute	PD-L1 positivity is associated with improved response to ICI
Immune cell infiltration	Que et al. [61]	2017	Retrospective	163 STS tumors from a single institute	FOXP3 + tumor infiltrating Tregs associated with age of higher tumor stage, higher tumor grade, and tumor depth
	Toulmonde et al. [56]	2018	Phase 2 clinical trial	57 patients with advanced unresectable STS	Strong infiltration by M2 macrophages in majority of cases. UPS had the highest proportion of M2 macrophages to total macrophages
	Dancsok et al. [58]	2019	Retrospective	1242 sarcoma patients from multiple institutes	Genomically-complex sarcomas had higher TILs than translocation-associated sarcomas. Prior exposure to radiotherapy was associated increased immune infiltrates. Higher lymphocytic infiltration was associated with better OS among genomically complex sarcomas. Expression of PD-1 and CD56 were associated with worse OS.
	Dancsok et al. [53]	2020	Retrospective	1242 sarcoma patients from multiple institutes	UPS and other non-translocation sarcomas had higher TAMs than translocation-associated sarcomas. TAMs outnumbered TILs across nearly all sarcoma types. M2 macrophages outnumbered M1 macrophages.
	Chen et al. [52]	2020	Retrospective	101 STS patients from a single institute	UPS TME is dominated by tumor associated macrophages and is abundant in CD8 + T cells with high PD-1 expression
	Wustrack et al. [60]	2021	Prospective and retrospective	15 freshly resected UPS tumors (prospective) and 36 archival UPS tumors (retrospective) from a single institute	Negative correlation between tumor-infiltration CD8 + T cells and UPS tumor size. Positive correlation between tumor-infiltrating CD8 + T cells and OS
	Guegan et al. [62]	2024	Prospective	47 UPS patients from multiple institutes	High immune infiltration, particularly CD8 + T cells and CD20 + B cells, predicts poor response to neoadjuvant chemotherapy in UPS
	Subramanian et al. [63]	2024	Retrospective	STS patients form multiple institutes	A sarcoma ecotype defined by tumor-associated macrophages and epithelial-like malignant cells predicts response to immune-checkpoint inhibition but not chemotherapy
Tertiary lymphoid structures	Petitprez et al. [31]	2020	Retrospective	STS patients from multiple institutes	An immune-high TME phenotype characterized by the presence of TLS and B cells was prognostic of survival and response to ICI
	Italiano et al. [30]	2022	Phase II clinical trial	Metastatic/nonresectable sarcoma patients from multiple institutes	Cohort of patients selected for intratumoral TLS presence had an ORR of 30% to ICI. Unselected patients had an ORR of 2.4%
	Roland et al. [73]	2024	Phase II clinical trial	Patients with surgically resectable UPS or DDLPS	Intratumoral TLS and B cells were associated with survival. Neoadjuvant ICI may stimulate formation of intratumoral TLS.

## Immune Checkpoint Inhibition for UPS/MFS

Response rates of high risk and/or advanced STS to chemotherapy are low, with an ORR of between 11–18% [74, 75]. Moreover, systemic chemotherapy often causes dose limiting toxicities, especially among older patients who make up the majority of UPS/MFS cases [76]. With the success of ICI therapy across multiple solid tumors, ICI is emerging as a promising treatment option for STS [77–83]. In addition to potentially being a more effective treatment, ICI is associated with fewer serious adverse events than chemotherapy, which may allow it to be offered to patients who are unable to tolerate chemotherapy [84]. Several clinical trials have been completed or are underway testing ICI in STS and, to date, UPS and MFS have been two of the most responsive STS subtypes to ICI therapy [38, 85, 86].

### ICI Monotherapy

The first clinical trial to demonstrate efficacy of ICI in STS was the SARC028 phase II clinical trial, which tested pembrolizumab (PD-1 inhibitor) in patients with metastatic or unresectable UPS, DDLPS, leiomyosarcoma (LMS), synovial sarcoma (SS), Ewing sarcoma, osteosarcoma, and chondrosarcoma [8]. 18% of patients with STS had an objective response, including four of ten patients with UPS (1 CR and 3 PR). Due to the encouraging signal for UPS, an expansion cohort was enrolled with 30 additional UPS patients for a total of 40 patients [72]. The ORR of the entire UPS cohort was 23% (9/40), with an additional 5/30 PRs observed in the expansion cohort. The median progression-free survival (PFS) for the entire UPS cohort was 3 months and the median OS was 12 months. Although the ORR of pembrolizumab in UPS was below the 25% considered clinically meaningful, it nevertheless demonstrated the potential activity of ICI in UPS.

Despite early promising results, clinical trials testing ICI monotherapy in UPS and MFS have yielded poor results overall. In the Alliance A091401 phase II clinical trial, which tested nivolumab (anti-PD-1) or nivolumab plus ipilimumab (anti-CTLA-4) in metastatic or unresectable STSs, nivolumab monotherapy resulted in an ORR of 5% (2/38), a median PFS of 1.8 months, and median OS of 10.7 months [87]. No UPS or MFS patients experienced a response. In another phase II study, which tested nivolumab monotherapy in advanced/recurrent STS, uterine cervical cancer, and uterine corpus cancer, STS patients (including 5 UPS patients) had an ORR of 0% and median PFS of 1.4 months [88]. These data have led to a shift toward the combination of ICI with another immunotherapy, chemotherapy, small molecular inhibitor, and/or radiation therapy

### ICI Combined with Another Immunotherapy

Although nivolumab monotherapy proved to be ineffective in the Alliance A091401 trial, combination of nivolumab and ipilimumab was more efficacious, demonstrating an ORR of 16%, with responses occurring in UPS, MFS, uterine LMS, and non-uterine LMS [87]. The median PFS was 4.1 months and the median OS was 14.3 months. An expansion cohort reaffirmed these outcomes, with combination of nivolumab and ipilimumab in UPS patients resulting in an ORR 16.6%, a PFS of 2.7 months, and an OS of 15.2 months [89]. A similar phase II clinical trial by Somaiah et al., which tested the combination of durvalumab (anti-PD-L1) and tremelimumab (anti-CTLA-4) in a variety of advanced or metastatic sarcoma subtypes, demonstrated an ORR of 14%, a median PFS of 2.8 months, and a median OS of 21.6 months [90]. In UPS, one out of five of patients had a PR.

### ICI Combined with Chemotherapy

ICI combined with chemotherapy has been shown to have increased efficacy in STS [85, 86]. In a phase I/II clinical trial by Pollack et al., which tested the combination of pembrolizumab and doxorubicin in patients with advanced anthracycline-naive sarcomas, the ORR was 13%, median PFS was 8.1 months, and median OS was 27.6 months [91]. Notably, two out of three patients with UPS had a durable PR. In a very similar phase II clinical trial by Livingston et al., patients with unresectable or metastatic anthracycline naïve STS were treated with a combination of pembrolizumab and doxorubicin [92]. The ORR was substantially higher at 36.7%; however, median PFS and median OS were lower at 5.7 months and 17 months respectively. The authors noted that the differences in outcomes between their trial and that of Pollack et al. may have been due to the variability in sarcoma subtypes enrolled, increased proportion of patients receiving the combination of pembrolizumab and doxorubicin as first-line systemic treatment, and the doxorubicin doses received. Notably, all UPS patients enrolled (4/4) experienced a PR. Pembrolizumab combined with doxorubicin versus doxorubicin alone for aggressive, poorly differentiated sarcomas is now being tested in a nationwide phase III clinical trial (NCT06422806) [93]. 

Another combination that has shown clinical activity is that of ipilimumab, nivolumab, and trabectedin, demonstrated in the SAINT phase I/II trial [94]. In this trial, patients with unresectable or metastatic STS including UPS and MFS received each of these drugs in combination as first-line therapy. The ORR was 25.3%, median PFS was 6.7 months, and median OS was 24.6 months. Notably, six patients experienced CR, including two UPS patients and one MFS patient.

The combination of sintilimab (anti-PD-1), doxorubicin, and ifosfamide has also shown efficacy in STS [95]. In the phase II trial reported by Liu et al., treatment naïve patients with unresectable or metastatic UPS, SS, myxoid liposarcoma, and DDLPS were treated with the combination. The ORR was an unprecedented 68.3%, with seven out of 8 UPS patients experiencing a response. Median PFS and OS were 9.0 months and 19.9 months respectively. Confirmation of this outcome in a phase III trial may lead to the adoption of this treatment combination as the gold standard for advanced STS [6]. However, there are serious concerns about the toxicity associated with combining immunotherapy with two cytotoxic agents.

As discussed above, retrospective analysis of the SARC028 trial by Petitprez et al. and Keung et al. provided the rationale for the PEMBROSARC trial to include a new cohort consisting of patients with intratumoral TLS present on biopsy [30]. Previously, the PEMBROSARC trial showed that the combination of pembrolizumab and cyclophosphamide had limited activity in STS [56]. In this trial, patients with advanced nonresectable or metastatic STSs received pembrolizumab combined with low dose cyclophosphamide. Patients from the TLS-positive cohort had a significantly longer 6-month NPR and ORR at 40% and 30% respectively, compared to non-selected patients at 4.9% and 2.4% respectively. Median PFS and OS were also significantly longer in the TLS-positive cohort, at 4.1 months and 18.3 months respectively, compared to non-selected patients, at 1.4 months and 14.3 months respectively. However, no UPS or MFS patient in the TLS-positive cohort had a response.

Not all clinical trials combining ICI with chemotherapy have shown efficacy. In the phase Ib TRAMUNE study, patients with advanced pretreated STS (including 2 UPS patients) were treated with a combination of trabectedin and durvalumab [96]. The ORR was 7%, the 6-month PFS was 28.6%, and the 1-year PFS rate was 14.3%.

Finally, high-dose local administration of chemotherapy combined with ICI for STS of the extremities has shown early signals of efficacy. A case report of 2 patients with recurrent MFS treated with isolated limb infusion of chemotherapy combined with pembrolizumab demonstrated excellent responses, with one patient having a significant PR lasting 6 months and the other patient having a CR lasting 2 years [97]. These promising responses have prompted the initiation of a phase II clinical trial investigating the combination [98].

### ICI Combined with Tyrosine Kinase Inhibitor (TKI)

Although the combination of ICI and TKI has demonstrated activity in certain sarcoma subtypes, it has shown varying efficacy in UPS and MFS [86]. In the IMMUNOSARC trial, which treated patients with advanced STS with a combination of nivolumab and sunitinib (multi-kinase inhibitor), the ORR was 21%, the 6-month PFS rate was 48%, and the median OS was 24 months [99]. However, no UPS patients had a response. Similarly, in a phase II clinical trial that tested the combination of axitinib plus pembrolizumab in patients with advanced or metastatic sarcomas, no UPS patients had a response [100]. This combination did show efficacy in other sarcoma subtypes, with an ORR of 25%, a median PFS of 4.7 months, and median OS of 18.7. Another phase II trial that combined cabozatinib with nivolumab and ipilimumab for patients with metastatic STS resulted in an ORR of 11%, median PFS of 5.4 months, and median OS of 22.6 months for the combination treatment [101]. No responses were observed in patients with UPS or MFS.

Other clinical trials have shown that the combination of ICI with TKI has benefit in UPS and MFS. In a pilot study combining pembrolizumab and lenvatinib in advanced pretreated sarcoma, two out of five UPS patients experienced a PR (20% ORR), and the median PFS for the five patients was 25 weeks [102]. The median OS was not reached. In a phase II clinical trial that combined durvulamab and pazopanib in patients with metastatic and/or recurrent STS, the ORR was 30% and median PFS was 7.7 months [103]. Four UPS and four MFS patients were included in the trial. Of the four UPS patients, three had a PR while one had stable disease (near PR). However, none of the MFS patients had a response. Correlative analysis showed that patients with high B cell infiltration in their tumor had a longer PFS and better response to treatment, consistent with the findings of Petitprez et al. [31, 103].

### ICI Combined with Radiation Therapy

The combination of ICI with radiation therapy is thought to release tumor antigens and enhance the effect of ICI, with clinical trials of this strategy showing promise in STS [104–106]. In an ongoing phase II clinical trial by Roland et al. (NCT03307616), patients with resectable UPS were treated with a combination of nivolumab, ipilimumab, and radiotherapy prior to surgical resection [73, 107]. 89% of UPS patients experienced a pathologic response to therapy (percent hyalinization), while 20% of UPS patients had a PR on imaging. Survival outcomes were excellent, with 78% relapse-free survival and 90% OS at 24 months follow-up. As discussed earlier, this study confirmed previous findings that intratumoral B cells and TLS are associated with increased survival. A recent landmark trial that demonstrated the efficacy of combining ICI with radiotherapy was the SU2C-SARC032 study, which compared neoadjuvant pembrolizumab plus radiotherapy followed by surgery and adjuvant pembrolizumab (experimental group) with neoadjuvant radiotherapy followed by surgery (control group) in patients with localized high-risk STS (UPS, DDLPS, pleomorphic LPS) [9]. This was the first completed randomized clinical trial of adding ICI to radiation therapy and surgery in patients with high-risk, localized STS of the extremity. Two-year disease-free survival was 68% in the experimental group and 52% in the control group, demonstrating the potentially synergistic effect of neoadjuvant ICI and radiation therapy. These results support the addition of ICI to preoperative radiation therapy and surgery for high-risk, resectable UPS/MFS of the extremity or limb girdle. Please refer to Table 2 for a selected list of ICI clinical trials that include patients with UPS and MFS.

**Table 2:** Select ICI clinical trials including UPS or MFS

Category	Study	Year	Phase	Intervention	Population	*N*	Response	Survival
ICI monotherapy	Tawbi et al. (SARC028) [8]	2017	II	Pembrolizumab	Metastatic or surgically unresectable sarcoma	86, including 10 UPS	STS: ORR 18% Bone sarcoma: ORR 5% UPS: ORR 40% (1 CR, 3 PR)	STS: mPFS 18 week mOS 49 weeks Bone sarcoma: mPFS 8 week mOS 52 weeks UPS: mPFS 30 weeks mOS not reached
	D’Angelo et al. (Alliance A091401) [87]	2018	II	Nivolumab	Metastatic or surgically unresectable sarcoma	43, including 5 UPS	All: ORR 5% (2 PR) No responses in UPS/MFS	All: mPFS 1.7 months mOS 10.7 months
	Burgess et al. (SARC028 expansion cohorts) [72]	2019	II	Pembrolizumab	Metastatic or surgically unresectable UPS and LPS	80, including 40 UPS	UPS: ORR 23% (2 CR, 7 PR) LPS: ORR 10% (4PR)	UPS: mPFS 3 months mOS 12 months LPS: mPFS 2 months mOS 13 month
	Tamura et al. [88]	2019	II	Nivolumab	Advanced or recurrent uterine corpus cancer, uterine cervical cancer, and STS that is unresectable	64, including 2 MFS and 1 UPS	STS: ORR 0%	STS: mPFS 1.4 months mOS NE
	Seligson t. al (Alliance A091401 expansion cohorts) [89]	2024	II	Nivolumab	Locally advanced, unresectable, or metastatic GIST, UPS, or DDLPS	39, including 10 GIST, 15 DDLPS, and 14 UPS	UPS: ORR 8.3% (1 PR) DDLPS: ORR 8.3% (1PR) GIST: ORR 0%	UPS: mPFS 1.4 months mOS 6.6 months DDLPS: mPFS 4.6 months mOS 8.1 months GIST: mPFS 1.5 months mOS 9.1 months
ICI + immunotherapy	D’Angelo et al. (Alliance A091401) [87]	2018	II	Nivolumab + ipilimumab	Metastatic or surgically unresectable sarcoma	42, including 6 UPS and 1 MFS	All: ORR 16% (6 PR) UPS: 3 PR MFS: 1 PR	All: mPFS 4.1 months mOS 14.3 months
	Somaiah et al. [90]	2022	II	Nivolumab + tremelimumab	Recurrent or metastatic sarcoma	62, including 5 UPS	All: ORR 12% UPS: ORR 20% (1 PR)	All: mPFS 2.8 months mOS 21.6 months
	Seligson et al. (Alliance A091401 expansion cohorts) [89]	2024	II	Nivolumab + ipilimumab	Locally advanced, unresectable, or metastatic GIST, UPS, or DDLPS	79, including 21 GIST, 29 DDLPS, and 29 UPS	UPS: ORR 16.6% (2 PR) DDLPS: ORR 16.6% (2 PR) GIST: ORR 0%	UPS: mPFS 2.7 months mOS 15.2 months DDLPS mPFS 5.5 months mOS 14.6 months GIST: mPFS 2.9 months mOS 12.2 months
ICI + chemotherapy	Pollack et al. [91]	2020	I/II	Pembrolizumab + Doxorubicin	Metastatic or unresectable anthracycline-naïve sarcoma	37, including 3 UPS	All: ORR 19% UPS: 66% (2 PR)	All: mPFS 8.1 months mOS 27.6 months
	Livingston et al. [92]	2021	II	Pembrolizumab + Doxorubicin	Metastatic or unresectable anthracycline-naive sarcoma	30, including 4 UPS	All: ORR 36.7% UPS: 100% (4 PR)	All: mPFS 5.7 months mOS 17 months
	Italiano et al. (PEMBROSARC) [30]	2022	II	Pembrolizumab + cyclophosphamide	Unresectable or metastatic STS	TLS-positive cohort: 30 including 4 UPS All-comers cohort: 41 including 4 UPS	TLS-positive cohort: ORR 30% (no responses in UPS/MFS) All-comers cohort: ORR 4.9%	TLS-positive cohort: mPFS 4.1 months mOS 18.3 months All-comers cohort: mPFS 1.4 months mOS 14.3 months
	Gordon et al. (SAINT) [94]	2023	I/II	Nivolumab + ipilimumab + trabectedin	Unresectable or metastatic STS	79, including 9 UPS and 4 MFS	All: ORR 25.3% UPS: ORR 22.2% (2 CR) MFS: ORR 50% (1 CR, 1 PR)	All: mPFS 6.7 months mOS 24.6 months
	Liu et al. [95]	2024	II	Sintilimab + doxorubicin + ifosfamide	Unresectable or metastatic UPS, SS, MLPS, DDLPS	46, including 20 UPS	All: ORR 68.3% UPS: ORR 65%	All: mPFS 9 months mOS 19.9 months
ICI + tyrosine kinase inhibitor	Wilky et al. [100]	2019	II	Pembrolizumab + axitinib	Advanced STS	33, including 5 UPS	All: ORR 25% (no responses in UPS)	All: mPFS 4.7 months mOS 18.7 months
	Martin-Broto et al. (IMMUNOSARC) [99]	2020	Ib/II	Nivolumab + sunitinib	Advanced STS	52, including 7 UPS	All: ORR 21% (no responses in UPS)	All: 6-month PFS 48% mOS 24 months
	Van Tine et al. [101]	2023	II	Nivolumab + ipilimumab + cabozantinib	Metastatic STS	69	All: ORR 11% (5 PR and 2 CR) MFS was among the responding histologies	All: mPFS 5.4 months mOS not reached
	Movva et al. [102]	2024	II	Pembrolizumab + lenvatinib	Unresectable or metastatic STS	46, including 10 UPS	UPS: ORR 25% (2 PR)	UPS: mPFS 25 weeks mOS not reached
	Cho et al. [103]	2024	II	Durvalumab + pazopanib	Metastatic or recurrent STS	46, including 4 UPS and 4 MFS	All: ORR 30.4% UPS: ORR 75% (3 PR ) MFS: ORR 0%	All: mPFS 7.7 months mOS not reached
ICI + radiotherapy	Roland et al. [73]	2024	II	Neoadjuvant nivolumab *or* nivolumab/ipilimumab + radiotherapy	Resectable extremity/truncal UPS	10 UPS	UPS: Response on imaging 20% (2 PR) Pathologic response 89%	UPS: RFS at 24 months 78% OS at 24 months 90%
	Mowery et al. (SU2C-SARC032) [9]	2024	II	Neoadjuvant pembrolizumab + radiotherapy then surgery + postoperative pembrolizumab (experimental group) vs. neoadjuvant radiotherapy then surgery (control group)	UPS/MFS, DDLPS, pleomorphic LPS	127 (control = 63; experimental = 64)	Not given	Experimental: 2-year DFS 67% Control: 2-year DFS 52%

### Other ICI Combinations

Other clinical trials have combined ICI with immunomodulators such as IL-2 agonists and oncolytic virotherapy, with varying degrees of success. A pilot study tested the combination of bempegaldesleukin, an IL-2 agonist, with nivolumab in patients with advanced or metastatic sarcomas [108]. Bempagaldesleukin was shown in previous studies to increase proliferation and activation of TILs in solid tumors, leading the investigators to hypothesize that it would enhance the efficacy of ICI in sarcoma. For UPS/MFS patients, the ORR was 20% (two out of ten patients experienced a PR), the median PFS was 2.4 months, and the median OS was 9.2 months.

Several clinical trials have tested the combination of ICI with oncolytic virotherapy. A phase II clinical trial by Kelly et al. tested tamilogene laherparepvec (T-VEC) – a modified human herpes simplex virus type I designed to lyse tumor cells that is FDA-approved to treat melanoma – with pembrolizumab in patients with locally advanced or metastatic sarcoma who had failed at least one line of therapy [109]. The ORR was an impressive 35% (7 out of 20 patients), the median PFS was 17.1 weeks, and the median OS was 74.7 weeks. PR was observed in two UPS patients and one MFS patient. An expansion cohort demonstrated impressive results in patients with cutaneous angiosarcoma, with an ORR of 71% (five out of seven patients) [110]. However, only one of ten UPS/MFS patients in the expansion cohort experienced a response.

Another phase II trial by Toulmonde et al. tested the combination of avelumab (anti-PD-L1), metronomic cyclophosphamide, and JX-594, an oncolytic vaccinia virus in patients with advanced “cold” STS characterized by an absence of TLS [111]. The investigators hypothesized that the combination of cyclophosphamide and JX-594 would have immunostimulatory effects that sensitized the tumors to ICI. While they found that treatment with JX-594 led to significant changes in the TME, including increased T cell infiltration, the clinical activity of this combination was disappointing, with only one PR out of fifteen patients enrolled. Median PFS was 1.8 months, and median OS was 10.5 months. One UPS patient was treated and had progressive disease.

## Other Immunotherapy Strategies

Aside from ICI, several other forms of immunotherapy for UPS/MFS are in clinical trials or preclinical development. These include adoptive cell therapies, antibody-drug conjugates, and immunomodulators.

### Adoptive Cell Therapies

The first engineered cell therapy FDA-approved for solid tumor is afamitresgene autoleucel (afami-cel), a MAGE-A4 directed T-cell receptor (TCR)-based cellular therapy for synovial sarcoma, which was approved in August 2024. MAGE-A4 is a cancer testis-antigen highly and nearly ubiquitously expressed in synovial sarcoma, making it an excellent target. The SPEARHEAD-1 phase II trial showed that treatment with afami-cel in patients with heavily pretreated synovial sarcoma had an ORR of 37%, median PFS of 3.8 months, and a median OS of 15.4 months [112]. Another cancer testis-antigen highly expressed in synovial sarcoma is NY-ESO-1, and a phase I/II clinical trial testing a NY-ESO-1-targeted TCR therapy also demonstrated antitumor activity in synovial sarcoma [113]. Both NY-ESO-1 and MAGE-A4 are expressed to varying degrees in UPS and MFS. One study showed that NY-ESO-1 is positive in 35.3% (6/17) of MFS tumors and 11.1% (3/27) of UPS tumors, with positivity defined as greater than 50% of positive tumor cells and a staining intensity of “moderate” or “strong.” [114] Another study showed that MAGE-A4 is positive in 66.6% (6/9) MFS tumors and 60% (6/10) UPS tumors, albeit with a positivity defined as a lenient ≥ 5% of positive tumor cells [115]. Nevertheless, they show that on average, 37.8% of cells in MFS tumors and 34.2% of cells in UPS tumors were positive for MAGE-A4. These data suggest that a NY-ESO-1- or MAGE-A4-directed TCR therapy may be effective in a subset of UPS/MFS patients. To that end, a case report of a NY-ESO-1 positive UPS patient treated with autologous NY-ESO-1-specific TCR therapy showed initial tumor regression [116]. However the tumor eventually lost NY-ESO-1 expression, leading to disease progression.

Adoptive cell transfer with TILs is another strategy being pursued. Mullinax et al. showed that it is feasible to derive and expand TILs from several STS subtypes including UPS and MFS, and that these TILs have tumor-specific reactivity [117]. A pilot trial combined LTX3-15, an oncolytic peptide, and TILs in patients with advanced or metastatic STS (no UPS/MFS patients were included) [118]. Although a systemic immune response was induced in patients, the combination ultimately had limited clinical efficacy.

### Antibody-based Therapeutics

Antibody-drug conjugates (ADC) have shown significant efficacy in the treatment of various solid and hematologic malignancies [119]. The ideal target antigen for ADCs is one that is abundantly expressed on the surface of cancer cells and minimally expressed on normal tissues; such targets have been difficult to identify in UPS and MFS [120]. A phase I clinical trial tested ABBV-085, an ADC targeting LRRC15, in several cancers including UPS, with two out of ten UPS patients having a PR [121]. Although the 20% ORR in UPS was considered clinically significant, further development of ABBV-05 was discontinued. Another ADC currently in clinical trials for STSs is mecbotamab vedotin, which targets AXL, a receptor tyrosine kinase highly expressed several sarcoma subtypes including UPS and MFS [122]. In this phase II trial, patients with AXL-expressing advanced refractory sarcoma received either mecbotamab vedotin monotherapy or combined with nivolumab. Pollack et al. reported in 2024 that ORR is 3.5% in the monotherapy group and 4.5% in the mecbotamab vedotin + nivolumab combination group; the study is ongoing [123].

### Oncolytic Virotherapy

Oncolytic viruses are an emerging class of immunotherapy that exploit the innate ability of certain replication-competent viruses to infect and preferentially lyse tumor cells while leaving non-neoplastic cells intact [124]. They are often combined with other cancer treatment strategies designed to mediate tumor regression through alternative means. As discussed earlier, oncolytic virotherapy has been combined with ICI in STS clinical trials under the hypothesis that the virotherapy could trigger an immune response that potentiates the efficacy of ICI. Aside from combination with ICI, oncolytic virotherapy has been combined with radiotherapy. A phase IB/II clinical trial combined intratumoral T-VEC injection with external beam radiation therapy followed by surgery in patients with locally advanced STS of the extremities and trunk, including 13 UPS patients and 2 MFS patients [125]. Of the 30 patients treated, only one had a PR. The 2-year PFS was 57% and the 2-year OS was 77%.

An approach to improve the efficacy of oncolytic virotherapy plus radiation therapy was explored in a translational study by Floyd et al., which found that deletion of ATRX increased the sensitivity of sarcomas to T-VEC plus radiation therapy [126]. ATRX is a chromatin remodeling protein and tumor suppressor that is commonly altered in UPS and other sarcomas (up to 24%). The investigators found in STS patients with *ARTX* genomic alterations who did not receive radiotherapy, disease specific survival was significantly worse than STS patients without *ARTX* genomic alterations. However, among STS patients who received ionizing radiation, there was no significant difference in survival between those with *ARTX* genomic alterations and those without, suggesting that *ARTX* genomic alterations sensitize tumors to radiotherapy. Additionally, the investigators showed that in a mouse model of STS, those with *ATRX* deletion had a significantly better response to T-VEC plus radiotherapy than those that did not. Thus, *ATRX* mutation status in UPS and other sarcomas may be useful as a biomarker for response to T-VEC and/or radiotherapy.

## Preclinical Studies

### Immunomodulation of TLS

Given the strong association between the presence of TLS and ICI response, it is hypothesized that induction of TLS in immunologically “cold” tumors could impart sensitivity to ICI. Several strategies to induce TLS are being pursued in preclinical studies. This includes use of immunoregulators such as delivery of LIGHT or LTα to the TME to enhance infiltration of T cells [127–129], immune-stimulating agents such as agonists of stimulator of interferon genes (STING) [130] or agonistic CD40 antibodies [131]chemokines associated with lymphoid formation such as CXCL13 and CCL21 [132, 133], injection of driver cells such as stromal cells that act as lymphoid tissue organizer cells [64], repression of Tregs [134, 135], enrichment of T follicular helper cells [136], and oncolytic virotherapy [137]. No study has yet tested these strategies to induce the formation of TLS in the context of UPS/MFS. However, a recent study showed that intratumoral STING activation resulted in tumor regression in a preclinical model UPS, with analysis of the tumors showing an upregulation of lymphocytic markers and infiltration of cytotoxic T-lymphocytes [138]. Additionally, the study showed that combination therapy of a STING agonist with ICI showed a significant survival benefit over either therapy alone. However, no analysis was conducted on the effect of STING agonism on TLS formation.

### Cytokine-induced Killer (CIK) Cell

Another adoptive cell therapy strategy that has shown promise against UPS in preclinical studies is CIK cells. CIK cells are a heterogenous population of immune effector cells that exhibit both T cell and NK cell properties and possess MHC-unrestricted cytotoxicity against a broad range of tumor cells [139]. Several preclinical studies by Sangiolo et al. have shown that CIK cells are highly effective against STS. Using a UPS xenograft model, the group demonstrated that treatment with autologous CIK cells significantly delayed tumor growth [140]. In another study, the group demonstrated that CIK cells are effective against sarcoma stem cells resistant to chemotherapy and molecular targeted therapy, again using a UPS model [141]. In yet another study, the group demonstrated that CIK cells – redirected with a chimeric antigen receptor targeting CSPG4, a cell surface proteoglycan highly expressed on several STS subtypes including UPS and MFS – was better at controlling a STS xenograft models (including UPS) than untransduced CIK cells [142]. CIK cells have been used in clinical trials against melanoma, renal cell carcinoma, lung cancer, and colorectal cancer, but have yet to be tested against STS [143].

## Conclusions

UPS and MFS are the most common histological subtypes of STS in adults, and the efficacy of current systemic treatment options is limited. Although STS are generally considered immunologically cold tumors, UPS and MFS are among the most immunogenic sarcoma subtypes and have shown promising response to ICI in clinical trials. Particularly promising are clinical trials combining ICI with chemotherapy and radiation therapy. Combination of ICI with immunomodulators such as oncolytic virotherapy have also shown early promise and should be further investigated. The most promising singular biomarker of ICI response in UPS/MFS is TLS. However, only 30% of STS patients with TLS positivity responded to ICI, indicating that better biomarkers are still needed. Evaluation of a panel of biomarkers appears to be the best approach to predicting response of UPS/MFS to ICI. In addition to ICI, other immunotherapy approaches such as adoptive cell transfer and antibody drug conjugates may be effective against UPS/MFS. TCR therapy has been shown to be an effective approach in SS, but limited work has been done in UPS/MFS despite a subset of patients possessing targetable cancer/testis antigens. An ADC is currently in clinical trials.

In summary, immunotherapy is a promising treatment modality in UPS/MFS and should be carefully considered highly aggressive, unresectable, or relapse and refractory forms of the disease.

## Key References

- Tawbi HA, Burgess M, Bolejack V, et al. Pembrolizumab in advanced soft-tissue sarcoma and bone sarcoma (SARC028): a multicentre, two-cohort, single-arm, open-label, phase 2 trial. Lancet Oncol. 2017;18(11):1493-1501. 10.1016/S1470-2045(17)30624-1
  - This reference is of outstanding importance because it is the first clinical trial demonstrating the efficacy of ICI in UPS.
- Petitprez F, de Reyniès A, Keung EZ, et al. B cells are associated with survival and immunotherapy response in sarcoma. Nature. 2020;577(7791):556-560. 10.1038/s41586-019-1906-8
  - This reference is of outstanding importance because it is, in part, a correlative study of the SARC028 trial showing that a high density of B cells and TLS in STS correlates with response to ICI.
- Italiano A, Bessede A, Pulido M, et al. Pembrolizumab in soft-tissue sarcomas with tertiary lymphoid structures: a phase 2 PEMBROSARC trial cohort. Nat Med. 2022;28(6):1199-1206. 10.1038/s41591-022-01821-3
  - This reference is of outstanding importance because it is the first study demonstrating that STS patients selected for the presence of TLS have improved response to ICI compared to unselected patients.
- Mowery YM, Ballman KV, Hong AM, et al. Safety and efficacy of pembrolizumab, radiation therapy, and surgery versus radiation therapy and surgery for stage III soft tissue sarcoma of the extremity (SU2C-SARC032): an open-label, randomised clinical trial. *The Lancet*. 2024;404(10467):2053-2064. 10.1016/S0140-6736(24)01812-9
  - This reference is of outstanding importance because it is a landmark clinical trial that demonstrates that the addition of ICI to radiation therapy and surgery provides benefit to patients with localized STS of extremity or trunk.
- Subramanian A, Nemat-Gorgani N, Ellis-Caleo TJ, et al. Sarcoma microenvironment cell states and ecosystems are associated with prognosis and predict response to immunotherapy. *Nat Cancer*. 2024;5(4):642-658. 10.1038/s43018-024-00743-y
  - This study is of outstanding importance because it uses bulk transcriptomics and machine learning to describe a group of sarcomas termed sarcoma ecotype 3 with the best response to ICI. This finding suggests that the optimal prediction of response to of STS to ICI likely will likely require a panel of biomarkers rather than singular biomarkers.

## Data Availability

No datasets were generated or analysed during the current study.

## Abbreviations

UPS: Undifferentiated pleomorphic sarcoma
MFS: Myxofibroarcoma
STS: Soft tissue sarcoma
TME: Tumor microenvironment
TMB: Tumor mutation burden
CNA: Copy number alteration
OS: Overall Survival
ICI: Immune checkpoint inhibition
PD-L1: Programmed death-ligand 1
PD1: Programmed Death-1 Receptor
TIIC: Tumor infiltrating immune cells
NK: Natural Killer Cells
Treg: Regulatory T cell
MDSCs: Myeloid-derived suppressor cells
TAM: Tumor associated macrophages
TIL: Tumor infiltrating lymphocytes
SE3: Sarcoma ecotype 3
TLS: Tertiary lymphoid structure
ORR: Objective response rate
NPR: Non-progression rate
DDLPS: Dedifferentiated liposarcoma
LMS: Leiomyosarcoma
SS: Synovial Sarcoma
PFS: Progression-free survival
TKI: Tyrosine kinase inhibitor
CR: Complete response
PR: Partial response
T-VEC: Tamilogene laherparepvec
TCR: T-cell receptor
ADC: Antibody-drug conjugate
STING: Stimulator of interfron genes
CIK: Cytokine-induced killer
CAR: Chimeric antigen receptor
